# Plasma Biomarkers Differentiate Parkinson’s Disease From Atypical Parkinsonism Syndromes

**DOI:** 10.3389/fnagi.2018.00123

**Published:** 2018-04-27

**Authors:** Chin-Hsien Lin, Shieh-Yueh Yang, Herng-Er Horng, Che-Chuan Yang, Jen-Jie Chieh, Hsin-Hsien Chen, Bing-Hsien Liu, Ming-Jang Chiu

**Affiliations:** ^1^Department of Neurology, National Taiwan University Hospital, College of Medicine, National Taiwan University, Taipei, Taiwan; ^2^MagQu Co., Ltd., New Taipei City, Taiwan; ^3^Graduate Institute of Electro-Optical Science and Technology, College of Science, National Taiwan Normal University, Taipei, Taiwan; ^4^Graduate Institute of Brain and Mind Sciences, College of Medicine, National Taiwan University, Taipei, Taiwan; ^5^Graduate Institute of Biomedical Engineering, College of Medicine, National Taiwan University, Taipei, Taiwan; ^6^Graduate Institute of Psychology, National Taiwan University, Taipei, Taiwan

**Keywords:** Parkinson’s disease, atypical parkinsonism syndrome, α-synuclein, tau, p-Tau181, amyloid beta 42

## Abstract

**Objective:** Parkinson’s disease (PD) has significant clinical overlaps with atypical parkinsonism syndromes (APS), which have a poorer treatment response and a more aggressive course than PD. We aimed to identify plasma biomarkers to differentiate PD from APS.

**Methods:** Plasma samples (*n* = 204) were obtained from healthy controls and from patients with PD, dementia with Lewy bodies (DLB), multiple system atrophy, progressive supranuclear palsy (PSP), corticobasal degeneration (CBD), or frontotemporal dementia (FTD) with parkinsonism (FTD-P) or without parkinsonism. We measured plasma levels of α-synuclein, total tau, p-Tau181, and amyloid beta 42 (Aβ42) by immunomagnetic reduction-based immunoassay.

**Results:** Plasma α-synuclein level was significantly increased in patients with PD and APS when compared with controls and FTD without parkinsonism (*p* < 0.01). Total tau and p-Tau181 were significantly increased in all disease groups compared to controls, especially in patients with FTD (*p* < 0.01). A multivariate and receiver operating characteristic curve analysis revealed that a cut-off value for Aβ42 multiplied by p-Tau181 for discriminating patients with FTD from patients with PD and APS was 92.66 (pg/ml)^2^, with an area under the curve (AUC) of 0.932. An α-synuclein cut-off of 0.1977 pg/ml could separate FTD-P from FTD without parkinsonism (AUC 0.947). In patients with predominant parkinsonism, an α-synuclein cut-off of 1.388 pg/ml differentiated patients with PD from those with APS (AUC 0.87).

**Conclusion:** Our results suggest that integrated plasma biomarkers improve the differential diagnosis of PD from APS (PSP, CBD, DLB, and FTD-P).

## Introduction

Atypical parkinsonism syndrome (APS), including multiple system atrophy (MSA), progressive supranuclear palsy (PSP), corticobasal degeneration (CBD), dementia with Lewy body (DLB), and frontotemporal dementia (FTD) with parkinsonism (FTD-P), have a large clinical overlap with Parkinson’s disease (PD). However, the treatment response to levodopa and prognosis are distinct in patients with PD and APS (Bower et al., 1997; Elbaz et al., 2003). There is a need for biomarkers to differentiate PD from APS, particularly in the early stages when diagnosis is most difficult.

The neuropathologies of APS could be broadly classified into two clusters. Alpha-synucleinopathies are characterized by α-synuclein accumulations in neurons or glias, such as PD, DLB, and MSA. Tauopathies, including PSP, CBD, and FTD-P, are characterized by intraneuronal depositions of tau protein. Previous biomarker studies have used cerebrospinal fluid (CSF) samples to identify disease-specific markers, but the procedure is relatively invasive and cannot be widely used for monitoring disease progression (Magdalinou et al., 2014). Specific markers reflecting these different pathologies within the peripheral circulation could be used as a surrogate non-invasive biomarker. Recently, plasma level of neurofilament light chain, which reflects subcortical axonal damage, was noted to be elevated in patients with APS when compared to patients with PD (Laurens et al., 2015; Hansson et al., 2017). However, studies simultaneously analyzing the neuropathology-related biomarker candidates in plasma are lacking.

We previously detected increased plasma levels of amyloid beta 42 (Aβ42), tau, and α-synuclein in patients with Alzheimer’s disease (AD) and PD compared to controls, using an ultra-sensitive immunoassay with immunomagnetic reduction method (IMR) (Yang et al., 2011; Chiu et al., 2014; Lin et al., 2017). We therefore applied this IMR-based immunoassay to evaluate the potential diagnostic accuracy of a panel of four biomarkers—α-synuclein, total and phosphorylated tau (p-Tau181), and Aβ42 in the plasma of patients with PD and APS.

## Materials and Methods

### Study Participants

All participants were recruited from the National Taiwan University Hospital (NTUH), a tertiary referral center in Taiwan. We analyzed 204 plasma samples from patients with PD with normal cognition (PD-NC, *n* = 37), PD with mild cognitive impairment (PD-MCI, *n* = 29), PD or Parkinson’s disease dementia (PDD, *n* = 36; DLB, *n* = 6; PSP, *n* = 5; MSA, *n* = 22; CBD, *n* = 3), FTD-P (*n* = 6), FTD without parkinsonism (*n* = 25), and healthy individuals serving as controls (*n* = 35). PD was diagnosed according to the United Kingdom PD Society Brain Bank clinical diagnostic criteria (Hughes et al., 1992). PD-MCI was diagnosed according to the Movement Disorder Society (MDS) task force diagnostic criteria using the level I global cognitive function test (Litvan et al., 2012). MDS task force criteria also were used to diagnose PDD, with a Mini-Mental State Examination (MMSE) score of 25 or less as the cut-off for identifying significant cognitive impairment in PD patients, as well as impairment of instrumental activities of daily living (e.g., inability to manage finances and cope in social situations) (Emre et al., 2007). Patients who received a diagnosis of MSA met the consensus statement on the diagnosis of that disorder (Gilman et al., 2008). Patients who received a diagnosis of PSP met revised MDS criteria (Hoglinger et al., 2017), and patients with CBD were diagnosed in accordance with criteria revised by an international consortium (Armstrong et al., 2013). DLB was diagnosed according to the criteria of the fourth consensus report of the DLB Consortium (McKeith et al., 2017), and we used recently updated diagnostic criteria for FTD (Chare et al., 2014). The 35 controls underwent cognitive testing and neurologic examination by neurologists (C.-H. Lin and M.-J. Chiu), and individuals with cognitive impairment or motor dysfunction were not included as controls in the present study. Motor symptom severity was evaluated using the motor subscale of the Unified Parkinson’s Disease Rating Scale (UPDRS part III; Goetz et al., 2008) and Hoehn-and-Yahr (H–Y) staging (Hoehn and Yahr, 1967). This study was approved by the institutional ethics board committee of the National Taiwan University Hospital (NTUH 201605001RINA), and all participants provided written informed consent to participate in the study.

### Measurement of Plasma α-Synuclein, Total and Phosphorylated Tau (p-Tau181), and Aβ42

A total of 10 ml of venous blood was drawn from each participant and centrifuged (2500 × *g* for 15 min) within 1 h of collection. The blood was sampled at the time when the diagnoses were just made. We used the same methodology employed in our previous studies to assay the plasma levels of α-synuclein, total tau, p-Tau181, and Aβ42 (Yang et al., 2011, 2016, 2017; Chiu et al., 2014; Lin et al., 2017). In brief, the reagent for IMR consists of magnetic nanoparticles functionalized with monoclonal antibodies against α-synuclein (sc-12767; Santa Cruz Biotechnology), dispersed in phosphate-buffered saline (PBS; MF-ASC-0060, MagQu Co., Ltd.), pH 7.2. The tau reagent (MF-TAU-0060, MagQu Co., Ltd.) is a PBS solution that contains magnetic nanoparticles with a monoclonal antibody (T9450, Sigma) against human tau protein immobilized on their surfaces. The p-Tau181 reagent (MF-PT1-0060, MagQu Co., Ltd.) contains magnetic nanoparticles with a monoclonal antibody against tau protein phosphorylated at threonine residue 181, and the Aβ42 reagent (MF-AB2-0060, MagQu Co., Ltd.) contains magnetic nanoparticles coated with a monoclonal antibody against human Aβ42 protein. These reagents are superparamagnetic with a saturated magnetization of 0.3 emu/g. After mixing of the reagent and the tested plasma sample, each mixture was put into a superconducting-quantum-interference-device (SQUID)-based alternative current (AC) magnetosusceptometer (XacPro-S, MagQu Co., Ltd.) to determine the time-dependent AC magnetic susceptibility, which approximates the association between magnetic nanoparticles and targeted protein molecules in the plasma (Yang et al., 2016). The reduction in magnetic susceptibility resulting from the association between magnetic nanoparticles and the targeted protein molecule can be sensed by the high-Tc SQUID magnetometer and is referred to as the IMR signal. The IMR signal thus reflects the concentration of the targeted protein. Duplicate measurements were performed for IMR signals at each concentration of target protein.

### Statistical Analyses

Numerical variables are expressed as means ± standard deviations or medians with 95% confidence intervals (CIs). For variables following a Gaussian distribution, data were compared using two-tailed *t*-tests, and multiple comparisons were performed using analysis of variance (ANOVA). To control for possible confounders (e.g., age, sex, disease duration), analysis of covariance (ANCOVA) was used for group comparisons, including as covariates any demographic variable that differed significantly between groups. For variables not following a normal distribution, data were compared using the Mann–Whitney test, which is the non-parametric equivalent of the independent samples *t*-test, and the Kruskal–Wallis test was used for comparing three or more groups. Correlations between variables were graphically analyzed using the slope of the regression line including the 95% CI. The correlation between variables was explored with Spearman correlation analysis and the standardized correlation coefficient presented. Diagnostic accuracy was assessed using receiver operating characteristic (ROC) analysis. We performed all analyses with Stata software (StataCorp LLC, College Station, United States). A *p*-value < 0.05 was considered significant.

## Results

### Clinical Characteristics

The demographic data and plasma levels of the four markers for all 204 participants are summarized in **Table 1**. The age and disease duration were significantly higher in patients with PD-MCI and PDD than in other patient groups or in controls (*p* < 0.01 by ANOVA). Patients with FTD combined with parkinsonism or not were younger than PD and other parkinsonian patients (PSP, CBD, and MSA) (*p* < 0.01 by Kruskal–Wallis test). The MMSE scores were significantly lower in patients in the PDD and FTD groups compared to other groups and controls (*p* < 0.01 by Kruskal–Wallis test). Patients with APS, including MSA, PSP, CBD, and FTD-P, had significantly greater motor severity (UPDRS part III scores and H–Y stages) compared with the PD group (*p* < 0.01 by ANOVA).

**Table 1:** Clinical characteristics and plasma biomarker levels of all study participants in individual diagnostic groups.

	Controls (*n* = 35)	PD (*n* = 102)				DLB (*n* = 6)	MSA (*n* = 22)	PSP (*n* = 6)	CBD (*n* = 3)	FTD-P (*n* = 6)	FTD without P (*n* = 25)	*p*-value
									
		All PD (*n* = 102)	PD-NC (*n* = 37)	PD-MCI (*n* = 29)	PDD (*n* = 36)							
Age (years)	62.6 ± 9.7	67.25 ± 10.9	62.0 ± 10.5	66.5 ± 11.2	75.6 ± 9.1	63.0 ± 2.6	65.8 ± 7.4	66.7 ± 4.7	62.0 ± 11.3	58.0 ± 5.2	59.3 ± 7.5	<0.01**
Gender (male, %)	40.0	52.3	49.7	56.8	51.7	66.7	53.3	50.0	52.3	49.8	51.2	0.18
Disease duration (year)	N.A.	7.8 ± 5.2	4.6 ± 2.3	8.1 ± 6.3	9.2 ± 7.3	4.3 ± 2.5	5.2 ± 3.1	5.2 ± 2.0	2.5 ± 2.1	4.1 ± 2.8	3.2 ± 2.1	0.03*
MMSE	29.2 ± 0.8	25.7 ± 1.9	28.7 ± 0.9	25.3 ± 1.8	17.8 ± 5.4	26.3 ± 2.1	27.3 ± 1.2	25.3 ± 1.9	27.3 ± 2.1	19.4 ± 1.2	18.3 ± 2.1	<0.01**
Hoehn–Yahr stage (on)	N.A.	1.8 ± 0.7	1.7 ± 0.9	1.9 ± 0.8	2.7 ± 1.1	2.3 ± 1.4	4.2 ± 2.1	4.1 ± 1.9	3.0 ± 1.2	2.9 ± 1.3	N.A.	<0.01**
Hoehn–Yahr stage (off)	N.A.	2.5 ± 1.8	2.3 ± 1.2	2.4 ± 1.2	3.8 ± 1.9	2.9 ± 1.8	4.8 ± 2.4	4.3 ± 2.1	3.3 ± 1.4	3.2 ± 1.6	N.A.	<0.01**
UPDRS part III scores (on)	N.A.	17.3 ± 8.9	16.4 ± 9.8	16.2 ± 7.6	18.2 ± 9.8	13.2 ± 8.6	25.1 ± 10.2	34.2 ± 9.8	23.2 ± 10.3	20.2 ± 8.7	N.A.	<0.01**
UPDRS part III scores (off)	N.A.	27.6 ± 11.2	25.8 ± 10.2	25.1 ± 10.6	33.6 ± 12.6	22.0 ± 9.8	32.6 ± 10.1	36.8 ± 12.1	33.6 ± 12.6	29.6 ± 10.3	N.A.	<0.01**
α-synuclein (pg/ml)	0.05 ± 0.02	6.93 ± 5.87	0.99 ± 0.18	1.67 ± 0.24	13.17 ± 5.58	0.66 ± 0.13	0.71 ± 0.11	0.94 ± 0.20	0.64 ± 0.43	0.98 ± 0.22	0.05 ± 0.01	<0.01**
Total tau (pg/ml)	12.12 ± 0.96	20.32 ± 2.73	19.02 ± 1.25	20.27 ± 2.43	21.67 ± 1.92	16.15 ± 1.59	20.72 ± 1.78	18.94 ± 2.12	14.74 ± 0.97	24.14 ± 2.06	41.53 ± 1.10	<0.01**
Phospho-tau (pg/ml)	1.52 ± 0.18	3.98 ± 1.38	3.72 ± 0.27	4.09 ± 0.43	4.13 ± 0.30	2.89 ± 0.56	5.14 ± 0.43	3.83 ± 0.69	3.19 ± 0.17	6.79 ± 0.17	6.82 ± 0.32	<0.01**
Aβ42 (pg/ml)	15.48 ± 0.17	16.23 ± 0.73	16.25 ± 0.42	15.80 ± 0.44	17.01 ± 0.75	14.65 ± 0.49	16.96 ± 0.67	15.38 ± 0.35	15.27 ± 0.34	17.59 ± 0.83	18.42 ± 0.60	<0.01**


*PD, Parkinson’s disease; MCI, mild cognitive impairment; PDD, Parkinson’s disease dementia; DLB, dementia with Lewy body; MSA, multiple system atrophy; PSP, progressive supranuclear palsy; CBD, corticobasal degeneration; FTD-P, frontotemporal dementia with parkinsonism; FTD without P, frontotemporal dementia without parkinsonism features; MMSE, Mini-Mental State Examination; UPDRS, Unified Parkinson’s Disease Rating Scale; N.A., not available. Numbers are expressed as mean ± standard deviation. ^∗^*p* < 0.05; ^∗∗^*p* < 0.01. The *p*-values was analyzed by multiple comparisons which were performed using analysis of variance (ANOVA) and Scheffe’s test was applied for *post hoc* analysis.*

### Plasma Biomarker Levels in Different Disease Groups

Because age is a potential confounding factor for the examined plasma biomarker levels, we performed analysis of correlation to analyze individual plasma biomarker levels and age in all participants. Consistent with our previous studies (Chiu et al., 2014, 2017; Lin et al., 2017), we found no significant correlation between age and plasma level of α-synuclein (correlation coefficient *r* = 0.04, *p* = 0.59 by Spearman correlation analysis), p-Tau181 (correlation coefficient *r* = 0.06, *p* = 0.50 by Spearman correlation analysis), or Aβ42 (correlation coefficient *r* = 0.11, *p* = 0.20 by Spearman correlation analysis) (**Supplementary Figure S1**). There was a modest correlation between age and plasma level of total tau protein (correlation coefficient *r* = 0.17, *p* = 0.04 by Spearman correlation analysis) (**Supplementary Figure S1**).

**FIGURE 1:**
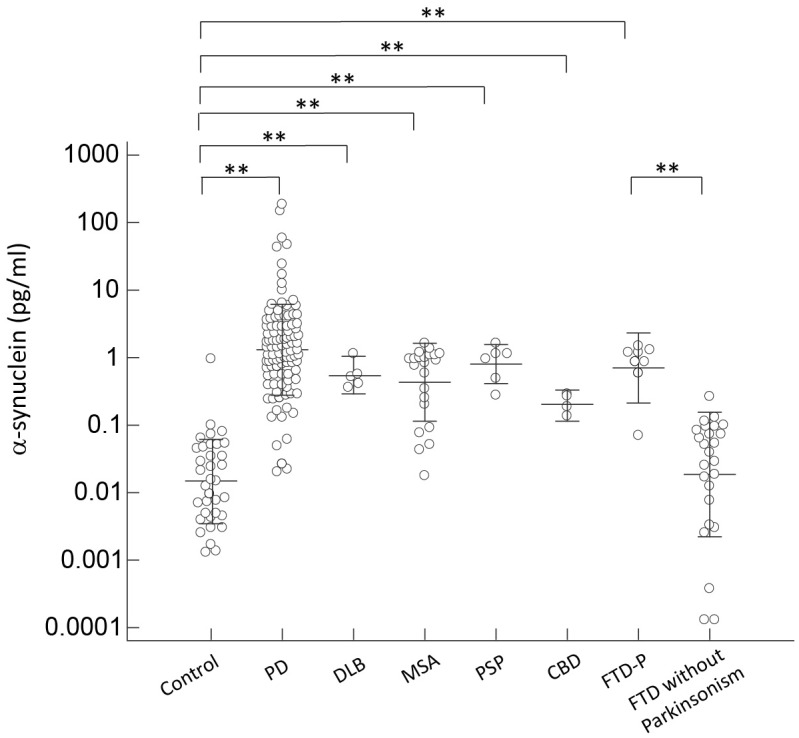
Plasma α-synuclein levels for all participants in the study. Plasma α-synuclein levels of normal controls and patients in different disease groups. The plasma α-synuclein level was significantly increased in patients with parkinsonism features compared to normal control participants and patients with FTD without parkinsonism (*p* < 0.001). The mean ± 1 standard deviation (SD) was illustrated as horizontal lines in each disease group. ^∗^*p* < 0.05; ^∗∗^*p* < 0.01.

To adjust for the potential confounding effects of age, we performed ANCOVA to compare individual biomarker levels in different diagnostic groups separately with age included in the model. We found that α-synuclein level was increased in patients with parkinsonian features, including PD, DLB, MSA, PSP, CBD, and FTD-P, when compared with controls and FTD patients without parkinsonism (*p* < 0.01 by Mann–Whitney test to compare individual disease group and controls; and *p* < 0.01 by Kruskal–Wallis test to compare all disease groups with parkinsonism features and controls or FTD without parkinsonism; **Figure 1**). Consistent with our previous study (Lin et al., 2017), the level of α-synuclein correlated with cognitive decline in PD patients in the sub-group analysis in PD patients, i.e., the α-synuclein level was 13.17 ± 5.58 in PDD, 1.67 ± 0.24 in PD-MCI, and 0.99 ± 0.18 in PD-NC (*r* = 0.56, *p* < 0.01 by Spearman correlation analysis, **Supplementary Figure S2A**). Consistently, the level of α-synuclein also correlated with scores of MMSE in PD patients (*r* = -0.35, *p* < 0.01 by correlation analysis, **Supplementary Figure S3A**). Of note, among FTD patients, patients with parkinsonism had significantly higher α-synuclein levels than patients without combined parkinsonism (0.98 ± 0.22 vs. 0.05 ± 0.01, *p* < 0.01; **Figure 1**). Among patients presenting with parkinsonian features, there was no difference between PD and APS (*p* = 0.53 by Kruskal–Wallis test to compare all disease groups with APS and PD).

**FIGURE 2:**
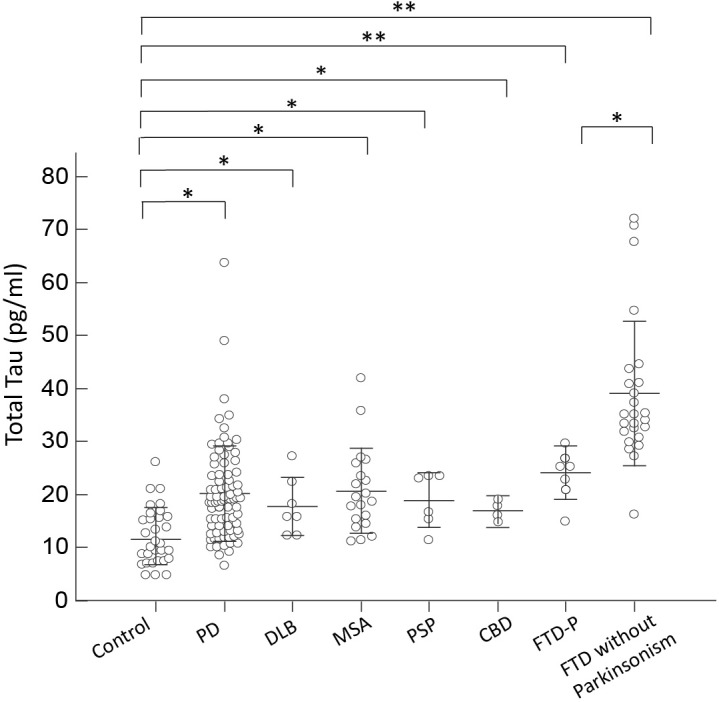
Plasma total tau levels for all participants in the study. Plasma total tau levels of normal controls and patients in different disease groups. The plasma total tau level was significantly increased in patients with FTD compared to normal control participants and patients with PD, DLB, MSA, PSP, or CBD (*p* < 0.001). The mean ± 1 standard deviation (SD) was illustrated as horizontal lines in each disease group. ^∗^*p* < 0.05; ^∗∗^*p* < 0.01.

For the neuronal injury marker total tau, we observed an increased plasma level in all disease groups, especially in patients with FTD, compared to controls (*p* < 0.01 by Kruskal–Wallis test to compare all disease groups and controls; **Figure 2**). Of note, among FTD patients, patients presenting with pure dementia without parkinsonian features had a much higher level of total tau than patients with combined parkinsonism (FTD-P, 41.53 ± 1.10 vs. 24.14 ± 2.06, *p* = 0.04 by Mann–Whitney test; **Figure 2**). Plasma total tau did not differ between PD and individual APS groups, including DLB, MSA, PSP, and CBD (*p* = 0.62 by Kruskal–Wallis test to compare all disease groups with APS and PD). Among PD patients, the expression levels of total tau did not correlate with either cognitive decline (*r* = 0.13, *p* = 0.25 by Spearman correlation analysis, **Supplementary Figure S2B**) or scores of MMSE (*r* = -0.21, *p* = 0.25 by Spearman correlation analysis, **Supplementary Figure S3B**).

**FIGURE 3:**
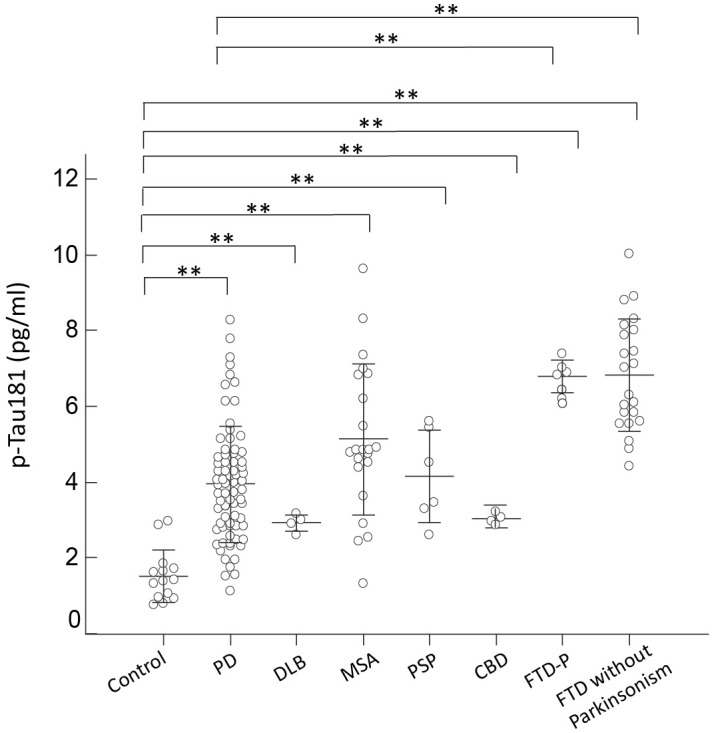
Plasma p-Tau181 levels for all participants in the study. Plasma p-Tau181 levels of normal controls and patients in the different disease groups. The plasma p-Tau181 level was significantly increased in patients with FTD compared to normal control participants and patients with PD, DLB, MSA, PSP, or CBD (*p* < 0.001). The mean ± 1 standard deviation (SD) was illustrated as horizontal lines in each disease group. ^∗^*p* < 0.05; ^∗∗^*p* < 0.01.

Consistent with the expression level of total tau, p-Tau181 was increased in all disease groups, especially in patients with FTD, who had a nearly twofold increased expression level compared to controls (*p* < 0.01 by Mann–Whitney test to compare individual disease group and controls; and *p* < 0.01 by Kruskal–Wallis test to compare all disease groups and controls; **Figure 3**). Among FTD patients, p-Tau181 levels did not differ between patients with or without parkinsonian features (6.79 ± 0.17 vs. 6.82 ± 0.32, *p* = 0.89 by Mann–Whitney test; **Figure 3**). Among PD patients, the expression levels of p-Tau181 did not correlate with either cognitive decline (*r* = 0.12, *p* = 0.32 by Spearman correlation analysis; **Supplementary Figure S2C**) or scores of MMSE (*r* = -0.28, *p* = 0.11 by Spearman correlation analysis; **Supplementary Figure S3C**). Notably, plasma levels of p-Tau181 differed between PD and FTD-P but were comparable with other APS disease groups (*p* < 0.62 by Mann–Whitney test to compare PD and FTD-P; **Figure 3**).

**FIGURE 4:**
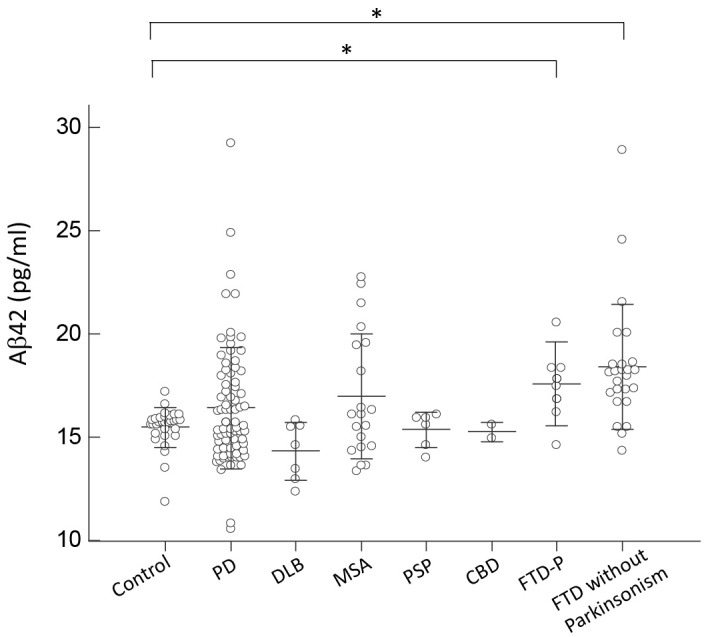
Plasma Aβ42 levels for all participants in the study. Plasma Aβ42 levels of normal controls and patients in different disease groups. The plasma Aβ42 level was significantly increased in patients with FTD compared to normal control participants and patients with PD, DLB, MSA, PSP, or CBD (*p* < 0.001). The mean ± 1 standard deviation (SD) was illustrated as horizontal lines in each disease group. ^∗^*p* < 0.05; ^∗∗^*p* < 0.01.

For Aβ42, only patients with FTD had an increased expression level of Aβ42 compared to controls and other disease groups (*p* = 0.45 by Kruskal–Wallis test to compare all disease groups and controls; **Figure 4**). Patients with PD or APS, including DLB, MSA, PSP, and CBD, had comparable levels of Aβ42 to those of controls (*p* = 0.57 by Kruskal–Wallis test to compare all disease groups with APS and PD; **Figure 4**). Among PD patients, the expression levels of Aβ42 did not correlate with either cognitive decline (*r* = 0.11, *p* = 0.34 by Spearman correlation analysis; **Supplementary Figure S2D**) or scores of MMSE (*r* = -0.17, *p* = 0.35 by Spearman correlation analysis; **Supplementary Figure S3D**).

### Diagnostic Accuracy of Integrated Plasma Markers in Differentiating PD and APS

**FIGURE 5:**
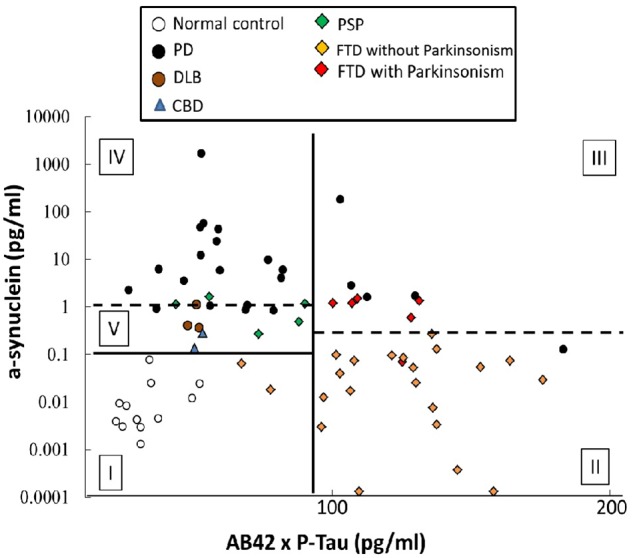
Diagnostic accuracy of plasma markers in differentiating PD and atypical parkinsonism syndromes. An ROC curve analysis was used to study the diagnostic accuracy when combining α-synuclein, p-Tau181, and Aβ42. The cut-off value of Aβ42 multiplied by p-Tau181 for discriminating patients with FTD from the parkinsonism groups (PD, DLB, PSP, and CBD) was 92.66 (pg/ml)^2^, resulting in 92.9 and 88.9% clinical sensitivity and specificity, respectively, and an AUC of 0.932 (vertical black line). Furthermore, among the FTD group, the cut-off value of α-synuclein was 0.1977 pg/ml for separating FTD-P from FTD without parkinsonism, for sensitivities of 88.3 and 95.5%, respectively, for FTD-P and FTD, and an AUC of 0.947 (right horizontal dashed line). In the patient groups with a value for Aβ42 multiplied by p-Tau181 of less than 92.66 (pg/ml)^2^, the cut-off for α-synuclein was 0.1038 pg/ml, which could differentiate patients with PD and atypical parkinsonism syndromes (DLB, PSP, and CBD) from controls (horizontal black line), with a sensitivity of 88.3% and a specificity of 95.5%; AUC = 1.00. Furthermore, 1.388 pg/ml α-synuclein could further discriminate PD from atypical parkinsonism syndromes (DLB, PSP, and CBD) with sensitivities of 73.9 and 90.0%, respectively, for PD and atypical parkinsonism syndromes (DLB, PSP, and CBD) and an AUC of 0.870 (left horizontal dashed line).

Although α-synuclein is the main pathognomonic protein in disorders of α-synucleinopathies, p-Tau181, and Aβ42 are also observed in patients with advanced stage of PD (Compta et al., 2011). p-Tau181, rather than total tau, is reported to associate with progression of PSP (Rojas et al., 2018) and motor severity of PD (Delgado-Alvarado et al., 2017). We therefore applied a ROC curve analysis was to study diagnostic accuracy in differentiating PD and APS when combining α-synuclein, p-Tau181, and Aβ42. PD patients with similar disease duration with patients of other disease group were enrolled into this analysis (**Supplementary Table S1**). For discriminating patients with FTD from patients with parkinsonism features (PD, DLB, PSP, and CBD), the cut-off value of Aβ42 multiplied by p-Tau181 (Aβ42 × p-Tau181) was 92.66 (pg/ml)^2^, resulting in 92.9% sensitivity and 88.9% specificity, and an area under the curve (AUC) of 0.932 (vertical black line, **Figure 5** and **Supplementary Figure S3**). Furthermore, among the FTD group, the cut-off value of α-synuclein was 0.1977 pg/ml which separated FTD-P from FTD without parkinsonism resulting in 88.3% sensitivity, 95.5% specificity, and an AUC of 0.947 (right horizontal dashed line in **Figure 5** and **Supplementary Figure S4**).

In the patient groups with an Aβ42 × p-Tau181 value less than 92.66 (pg/ml)^2^, combing the α-synuclein cut-off value of 0.1038 pg/ml could differentiate patients with PD and APS (DLB, PSP, and CBD) from controls (horizontal black line, **Figure 5** and **Supplementary Figure S4**), with 88.3% sensitivity, 95.5% specificity, and an AUC of 1.00. The further use of α-synuclein cut-off value of 1.388 pg/ml could discriminate PD from APS (DLB, PSP, and CBD) with a 73.9% sensitivity, 90.0% specificity, and an AUC of 0.870 (left horizontal dashed line, **Figure 5** and **Supplementary Figure S4**). In the above-mentioned algorithms, plasma levels of α-synuclein, p-Tau181, and Aβ42 contributed most to the accuracy of the differential diagnoses between disease groups. However, the distribution pattern of these markers in patients with MSA was diverse, and there was no significant difference between MSA and other parkinsonism syndromes using algorithms in this model (**Supplementary Figure S5**).

## Discussion

The results of this study demonstrate that integrated plasma biomarkers could be applied to differentiate PD from APS and FTD. Most specifically, plasma levels of Aβ42 and p-Tau181 improved the differential diagnosis of PD and APS from FTD. With the addition of α-synuclein, these integrated markers further differentiated PD from APS, except the subgroup of MSA. We have, for the first time, demonstrated that a panel of plasma markers targeting α-synuclein, p-Tau181, and Aβ42 could serve as surrogate biomarkers for assisting clinicians in diagnosing patients with parkinsonian features.

Previous studies have evaluated the ability of CSF level of α-synuclein or Aβ42 to differentiate among patients with α-synucleinopathy (PDD and DLB) and AD, but the results have been conflicting (Kasuga et al., 2010; Vranova et al., 2014). In this study, we observed a significantly higher plasma α-synuclein level in patients with PDD than in patients with DLB. Our findings are consistent with one previous study showing that CSF α-synuclein levels are higher in PDD than in DLB (Parnetti et al., 2011), although contradicted results were also reported (Mollenhauer et al., 2011). We speculate that these conflicting results may come from the heterogeneous cognitive status of the enrolled participants with PD or DLB. Our observations that plasma α-synuclein level is significantly higher in PDD than in PD-NC and DLB suggest that the underlying pathophysiology leading to α-synuclein depositions in cortical and nigral neurons in the cognitive dysfunction processes of PD and DLB may be different (Schulz-Schaeffer, 2010; Lin et al., 2017).

Several studies have suggested that CSF levels of total or phosphorylated tau may aid in differentiating PD from APS (Abdo et al., 2004; Sussmuth et al., 2010; Aerts et al., 2011). Several APS, including CBD, PSP, and FTD-P, are categorized as tauopathies, and one study has shown that the CSF level of total and phosphorylated tau is increased in CBD compared to PD and PSP (Aerts et al., 2011). However, this observation could not be replicated in later investigations (Parnetti et al., 2011; Hall et al., 2012; Vranova et al., 2014; Magdalinou et al., 2015), and our study also failed to demonstrate such elevations of total and phosphorylated tau in the plasma of patients with CBD or PSP. This disparity may possibly come from enrolment of less severely cognitively affected early-stage patients with CBD or PSP in our study, possibly reflecting less cortical involvement correlating with no elevation of plasma levels of total or phosphorylated tau. Another possible explanation may trace to the different isoforms of tau protein depositions in these disorders. Imbalances in the homeostasis of tau isoforms with three- (3R-tau) and four- (4R-tau) microtubule-binding repeat domains are important in neurodegenerative disease pathogenesis. In a normal adult brain, there are comparable levels of 3R-tau and 4R-tau, but in patients with PSP and CBD, the neurofibrillary tangles are predominantly 4R-tau, whereas Pick bodies in FTD are predominantly 3R-tau and neurofibrillary tangles in AD contain both 3R- and 4R-tau isoforms. One group had previously developed antibodies selective for the two isoforms and adapted an immuno-based procedure to detect CSF levels of each of them (Luk et al., 2012). A decrease in 4R-tau isoform was found in PSP and AD compared with CBD, PDD, and controls and without a difference in 3R-tau. We therefore hypothesize that 4R-tau could be used as a marker for differentiating PD, PSP, and CBD, but further studies exploring the expression levels of 4R-tau in the plasma are needed.

Of note, our results showed elevated plasma levels of total and phosphorylated tau (p-Tau181) in patients with FTD compared to PD and controls. Additionally, there was a significantly increased α-synuclein level in patients with FTD-P than in patients having FTD without parkinsonism. Several reports have described elevated CSF levels of total and phosphorylated tau in patients with FTD compared to patients with AD (Rosso et al., 2003; Pijnenburg et al., 2015; Kuiperij et al., 2017). Post-mortem studies have revealed that α-synuclein aggregates co-exist with tau pathology in patients with FTD-P (Yancopoulou et al., 2005). These observations and our findings reinforced the hypothesis that a crosstalk between tau and α-synuclein may work in concord to aggravate neurodegeneration in FTD-P (Moussaud et al., 2014). Our findings in the peripheral circulation reflect the brain pathology in patients with FTD-P, which suggests that plasma levels of total tau and p-Tau181 could be surrogate markers for FTD-P.

One of the major strengths of the present study was that we simultaneously assessed the diagnostic value of four plasma biomarkers (α-synuclein, total tau, p-Tau181, and Aβ42) that together reflect the major pathologies observed in PD and APS. To the best of our knowledge, the present study is the first to evaluate the potential diagnostic accuracy of combining several plasma biomarkers simultaneously in the differential diagnosis of these disorders. Another major advantage of this study is the use of the IMR-based method to detect plasma levels of targeted biomarkers, which manifests a low interference and high specificity for detecting plasma target proteins compared to ELISA (Yang et al., 2011, 2016). However, our study has some limitations. First, we did not investigate neurofilament light chain, which reflects subcortical axonal damage and was recently noted to be elevated in CSF and plasma of patients with MSA rather than PD (Sussmuth et al., 2010; Hansson et al., 2017). Second, the clinical diagnosis was not confirmed neuropathologically and is therefore susceptible to misclassification. However, the final diagnosis was based on thorough clinical and ancillary investigations (including nuclear imaging and neuropsychological assessment), after extensive clinical follow-up and according to international consensus criteria in a specialized movement disorder clinic. Third, the relatively small number of patients with PSP and CBD enrolled in the study may limit the extent to which our data can be extrapolated to all patients with atypical parkinsonism. Large cohort studies with a long follow-up period are needed to validate our results.

In summary, our findings suggest that integrated plasma levels of α-synuclein, total tau, p-Tau181, and Aβ42 improve the differential diagnosis of PD from APS (PSP, CBD, DLB, and FTD-P) and FTD. Longitudinal studies with a large number of patients are needed to evaluate the plausibility of these integrated plasma markers to assist in differentiating PD from APS, particularly in the early stages when diagnosis is most difficult.

## Author Contributions

Study concept and design: C-HL and M-JC. Acquisition of data: C-HL and M-JC. Analysis and interpretation of data: C-HL, S-YY, H-EH, C-CY, J-JC, H-HC, B-HL, and M-JC. Drafting the manuscript: C-HL. Critical revision of the manuscript for important intellectual content: C-HL and M-JC. Statistical analysis: C-HL. Study supervision: M-JC.

## Conflict of Interest Statement

S-YY, C-CY, H-HC, and B-HL are employees of MagQu Co., Ltd. and receive salaries from MagQu Co., Ltd. The other authors declare that the research was conducted in the absence of any commercial or financial relationships that could be construed as a potential conflict of interest.

## Supplementary Material

FIGURE S1 The correlation between plasma level of individual marker and age. **(A–D)** There was no significant correlation between age and plasma level of α-synuclein **(A)**, total tau **(B)**, p-Tau181 **(C)**, and Aβ42 **(D)**, except there was a modest correlation between age and plasma level of total tau protein **(B)** (correlation coefficient *r* = 0.17, *p* = 0.04).

FIGURE S2 The correlation between plasma level of individual marker and different cognitive status of PD patients. **(A–D)** The correlation between different cognitive status of PD patients and plasma level of α-synuclein **(A)**, total tau **(B)**, p-Tau181 **(C)**, and Aβ42 **(D)**.

FIGURE S3 The correlation between plasma level of individual marker and MMSE scores of PD patients. **(A–D)** The correlation between MMSE scores of PD patients and plasma level of α-synuclein **(A)**, total tau **(B)**, p-Tau181 **(C)**, and Aβ42 **(D)**.

FIGURE S4 An ROC curve analysis for diagnostic accuracy in differentiating PD and APS (DLB, PSP, CBD, and FTD-P), when combining α-synuclein, p-Tau181, and Aβ42.

FIGURE S5 Diagnostic accuracy of plasma markers in differentiating PD and APS which incorporated the disease group of MSA.

TABLE S1 Clinical characteristics of patients in individual diagnostic groups analyzed in **Figure 5**.
