# Supplementary figures and images for: Plasma Biomarkers Differentiate Parkinson’s Disease From Atypical Parkinsonism Syndromes
Source: Front Aging Neurosci. 2018 Apr 27;10:123. doi: 10.3389/fnagi.2018.00123 (PMC5934438; doi:10.3389/fnagi.2018.00123)

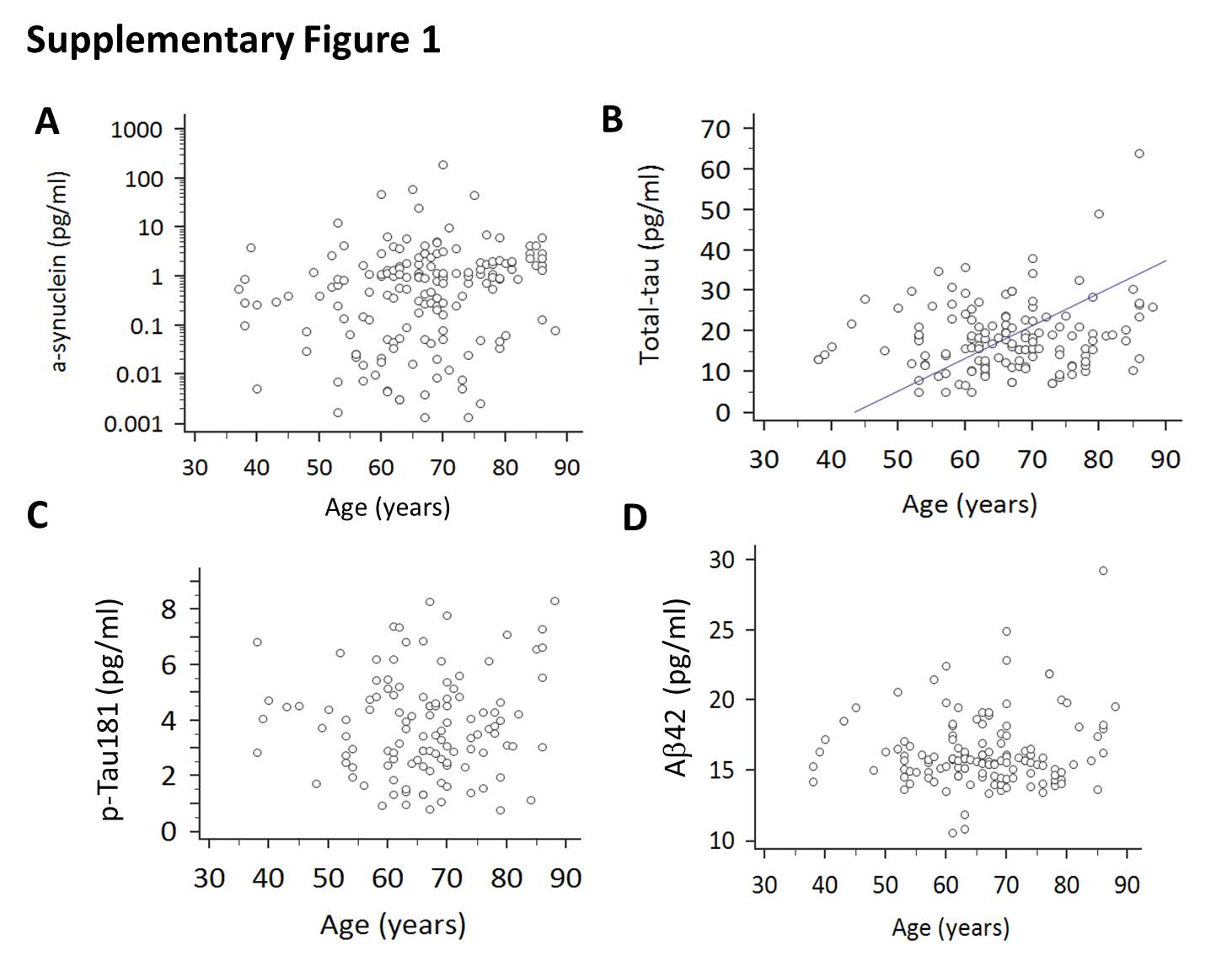

Supplement: FIGURE S1 — The correlation between plasma level of individual marker and age. (A–D) There was no significant correlation between age and plasma level of α-synuclein (A), total tau (B), p-Tau181 (C), and Aβ42 (D), except there was a modest correlation between age and plasma level of total tau protein (B) (correlation coefficient r = 0.17, p = 0.04). [file Image_1.JPEG]

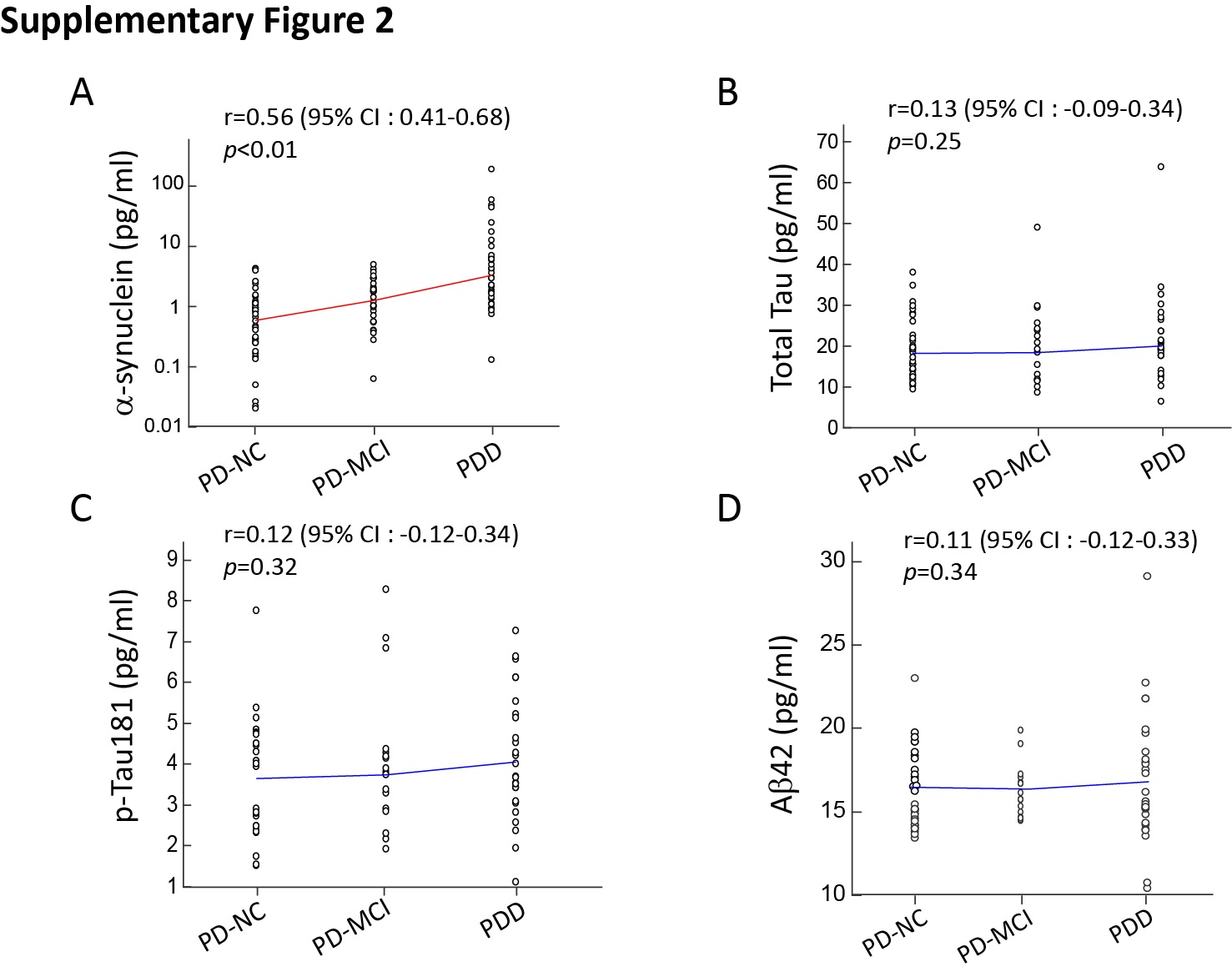

Supplement: FIGURE S2 — The correlation between plasma level of individual marker and different cognitive status of PD patients. (A–D) The correlation between different cognitive status of PD patients and plasma level of α-synuclein (A), total tau (B), p-Tau181 (C), and Aβ42 (D). [file Image_2.JPEG]

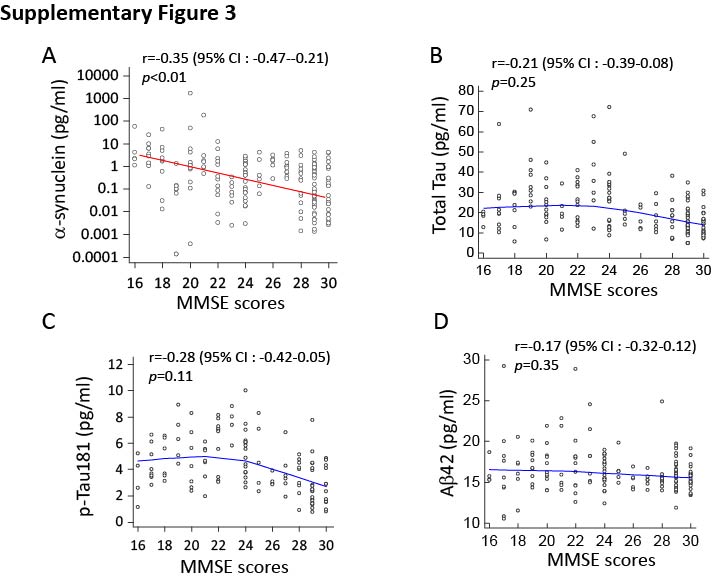

Supplement: FIGURE S3 — The correlation between plasma level of individual marker and MMSE scores of PD patients. (A–D) The correlation between MMSE scores of PD patients and plasma level of α-synuclein (A), total tau (B), p-Tau181 (C), and Aβ42 (D). [file Image_3.JPEG]

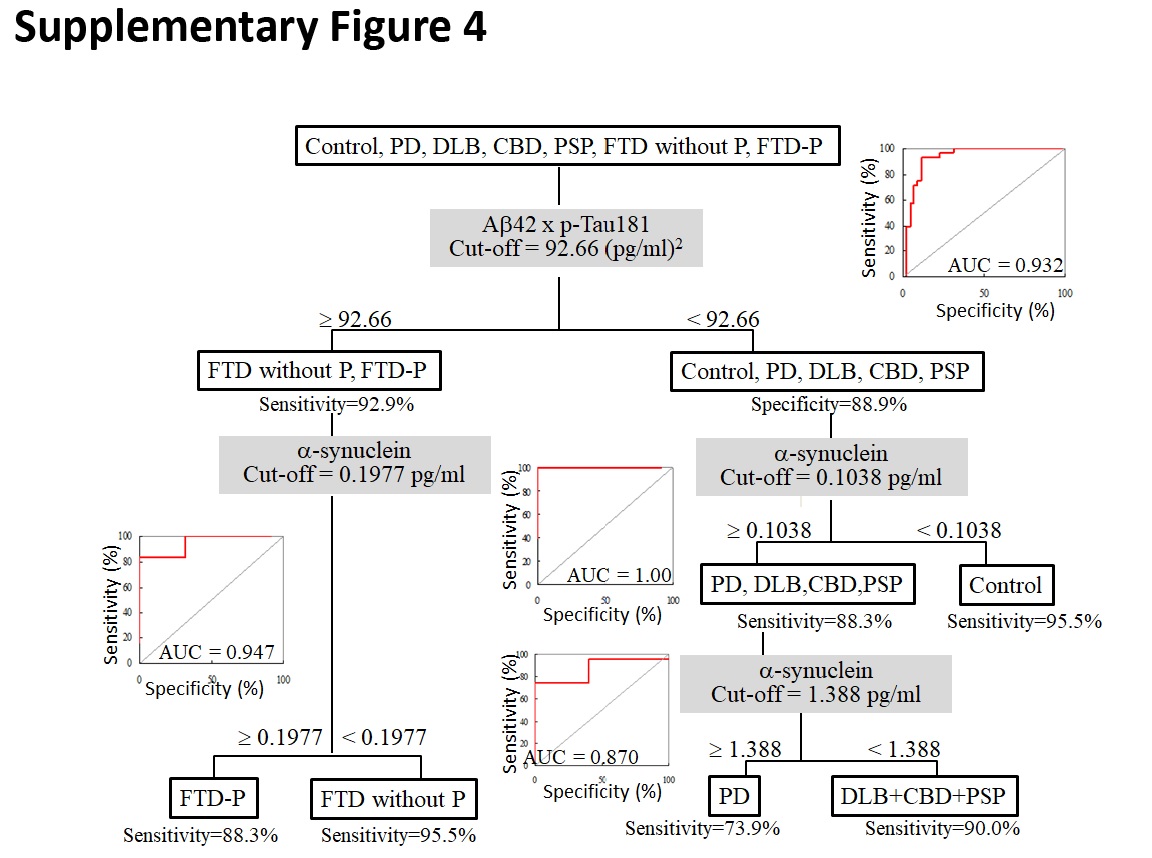

Supplement: FIGURE S4 — An ROC curve analysis for diagnostic accuracy in differentiating PD and APS (DLB, PSP, CBD, and FTD-P), when combining α-synuclein, p-Tau181, and Aβ42. [file Image_4.JPEG]

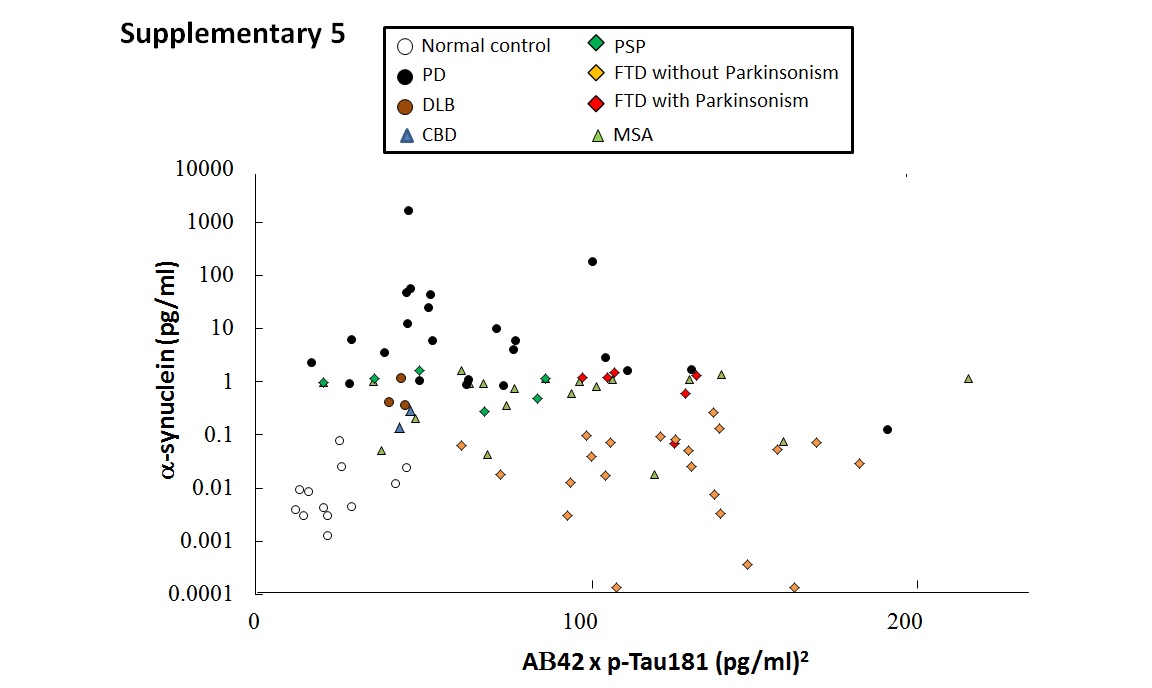

Supplement: FIGURE S5 — Diagnostic accuracy of plasma markers in differentiating PD and APS which incorporated the disease group of MSA. [file Image_5.JPEG]
