# Supplementary material for: Plasma Biomarkers Differentiate Parkinson’s Disease From Atypical Parkinsonism Syndromes
Source: Front Aging Neurosci. 2018 Apr 27;10:123. doi: 10.3389/fnagi.2018.00123 (PMC5934438; doi:10.3389/fnagi.2018.00123)
Supplement: TABLE S1 — Clinical characteristics of patients in individual diagnostic groups analyzed in Figure 5. [file Table_1.DOCX]

Supplementary Table 1. Clinical characteristics of patients in individual diagnostic groups analysed in Figure 5.

|  | Controls  (n=14) | PD  (n=28) | DLB (n=5) | PSP (n=5) | CBD (n=3) | FTD-P  (n=5) | FTD without P (n=20) | *p* value |
| --- | --- | --- | --- | --- | --- | --- | --- | --- |
| Age (years) | 60.1±8.7 | 62.2±10.5 | 62.5±2.7 | 64.7±5.2 | 62.0±11.3 | 59.3±6.2 | 59.8±8.1 | *P=*0.21 |
| Gender (M, %) | 50.0 | 53.5 | 60.0 | 60.0 | 67.7 | 60.0 | 55.0 | *p*=0.11 |
| Disease duration (y) | N.A. | 4.7±2.4 | 4.3±2.7 | 4.5±2.3 | 2.5±2.1 | 4.3±2.9 | 4.0±2.9 | *p*=0.09 |
| MMSE | 29.2±0.8 | 25.7±1.9 | 26.1±2.3 | 26.3±2.2 | 27.3±2.1 | 20.1±2.2 | 19.8±2.3 | *p*<0.05* |
| Hoehn-Yahr stage (on) | N.A. | 1.8±0.9 | 2.3±1.2 | 3.9±2.3 | 3.0±1.2 | 2.9±1.8 | N.A. | *p*<0.01** |
| Hoehn-Yahr stage (off) | N.A. | 2.3±1.5 | 2.8±1.9 | 4.2±2.3 | 3.3±1.4 | 3.2±1.8 | N.A. | *p*<0.01** |
| UPDRS part III scores (on) | N.A. | 15.3±9.1 | 12.8±8.9 | 33.2±9.3 | 23.2±10.3 | 19.5±8.8 | N.A. | *p*<0.01** |
| UPDRS part III scores (off) | N.A. | 27.6±11.2 | 22.7±8.8 | 36.5±9.5 | 33.6±12.6 | 27.8±10.2 | N.A. | *p*<0.01** |

PD, Parkinson’s disease; DLB, diffuse lewy body dementia; MSA, multiple system atrophy; PSP, progressive supranuclear palsy; CBD, corticobasal degeneration; FTD-P, frontotemporal dementia with parkinsonism; FTD without P, frontotemporal dementia without parkinsonism features; MMSE, mini-mental status examination; UPDRS, unified Parkinson's disease rating scale; N.A., not available. Numbers are expressed as mean±standard deviation. *P<0.05; **P<0.01. The p-values was analysed by multiple comparisons which were performed using analysis of variance (ANOVA) and Scheffe’s test was applied for post-hoc analysis.
